# Supplementary figures and images for: Excreted Trypanosoma brucei proteins inhibit Plasmodium hepatic infection
Source: PLoS Negl Trop Dis. 2021 Oct 29;15(10):e0009912. doi: 10.1371/journal.pntd.0009912 (PMC8580256; doi:10.1371/journal.pntd.0009912)

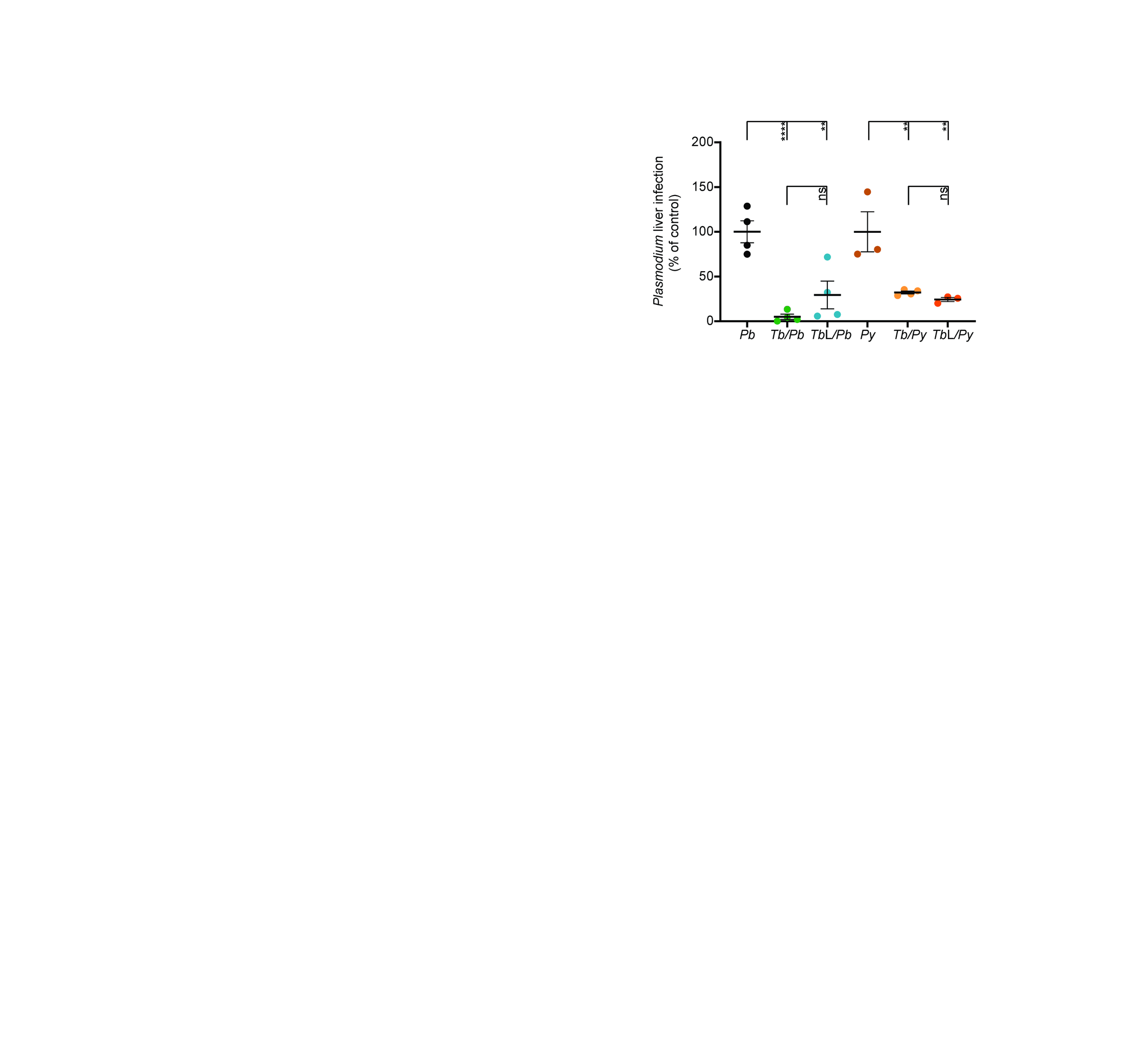

Supplement: S1 Fig — P. berghei (Pb) and P. yoelii (Py) liver infection load quantified by qRT-PCR 46 h after injection of P. berghei sporozoites into naïve mice (Pb–black symbols; Py–orange symbols), mice infected 5 days earlier with T. brucei (Tb/Pb–green symbols or Tb/Py–yellow symbols), or mice that received lysates of trypanosomes (TbL/Pb–blue symbols; or TbL/Py–dark orange symbols) 30 min prior to sporozoite inoculation. Symbols represent the individual values of each mouse in one independent experiment and error bars indicate the SEM. The one-way ANOVA with post-test Dunnett was employed to assess the statistical significance of differences between groups. ns, not significant, **p<0.01 and ****p<0.0001. (TIF) [file pntd.0009912.s001.tif]

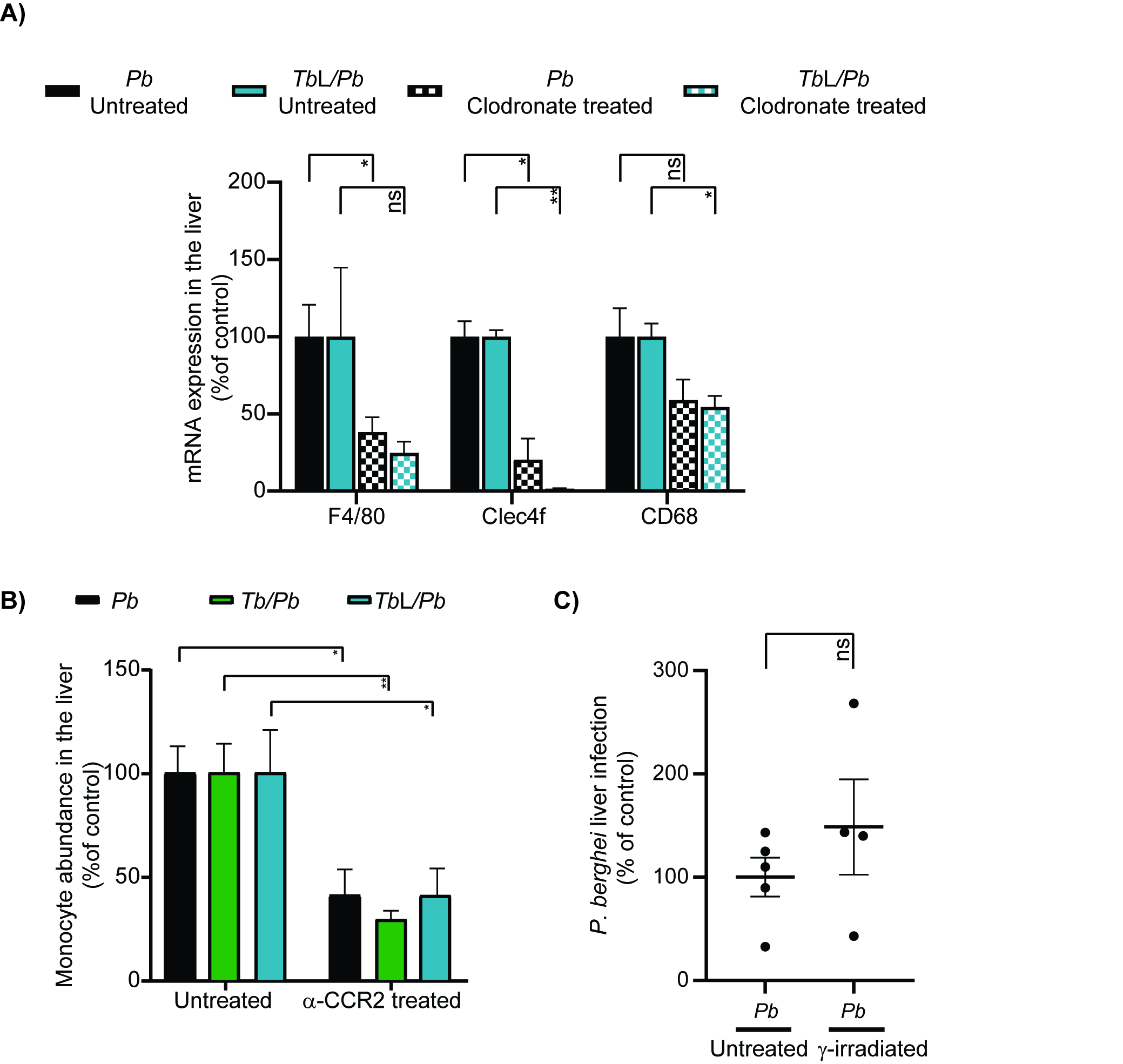

Supplement: S2 Fig — (A) Clec4f, F4/80 and CD68 gene expression quantification by qRT-PCR in the liver 46 h after injection of P. berghei sporozoites into naïve mice (Pb–black bars), or mice that received lysates of trypanosomes (TbL/Pb—blue bars) 30 min prior to sporozoite inoculation, non- or clodronate-treated 48 h prior to P. berghei infection. Bars represent the mean values of one independent experiment and error bars indicate the SEM. (B) Assessment of monocyte abundance in the liver by flow cytometry in the liver 46 h after injection of P. berghei sporozoites into naïve mice (Pb—black bars), infected 5 days earlier with T. brucei (Tb/Pb—green bars), or mice that received lysates of trypanosomes (TbL/Pb—blue bars) 30 min prior to sporozoite inoculation, administered or not with anti-CCR2. Bars represent the mean values of one independent experiment and error bars indicate the SEM. (C) P. berghei liver infection quantified by qRT-PCR 46 h after injection of P. berghei sporozoites into non- and γ-irradiated naïve mice, without normalization. Symbols represent the individual values of each mouse of one independent experiment and error bars indicate the SEM. For (A) and (B) The Mann-Whitney test was employed to assess the statistical significance of differences between experimental groups. ns, not significant, *p<0.05 and **p<0.01. For (C) an Unpaired t test was employed to assess the statistical significance of differences between experimental groups. ns, not significant. (TIF) [file pntd.0009912.s002.tif]

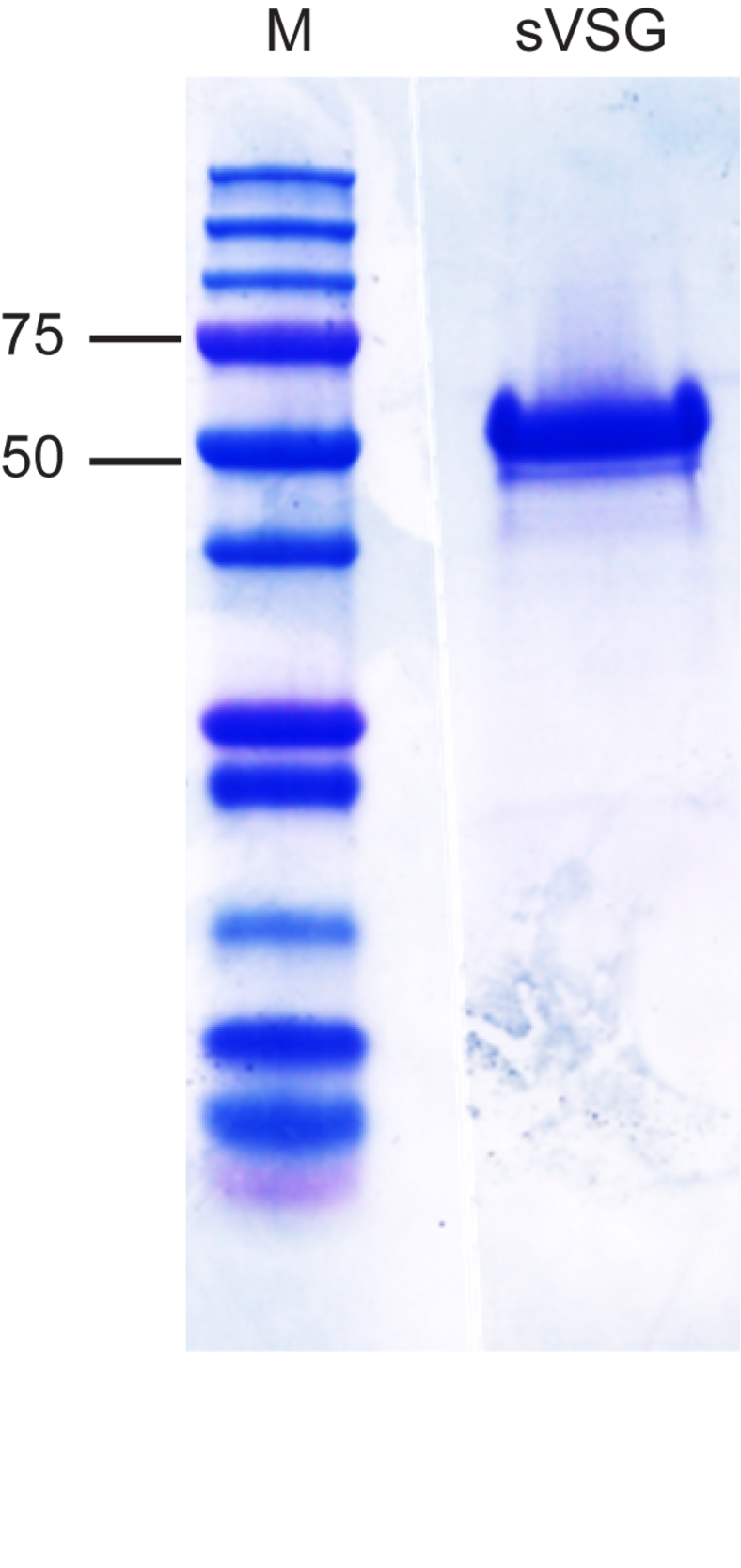

Supplement: S3 Fig — After purification of VSG2, 20 μL of purified sample were loaded on a 10% SDS-PAGE gel. The predicted molecular weight of VSG2 is ≈51 kDa. (TIF) [file pntd.0009912.s003.tif]

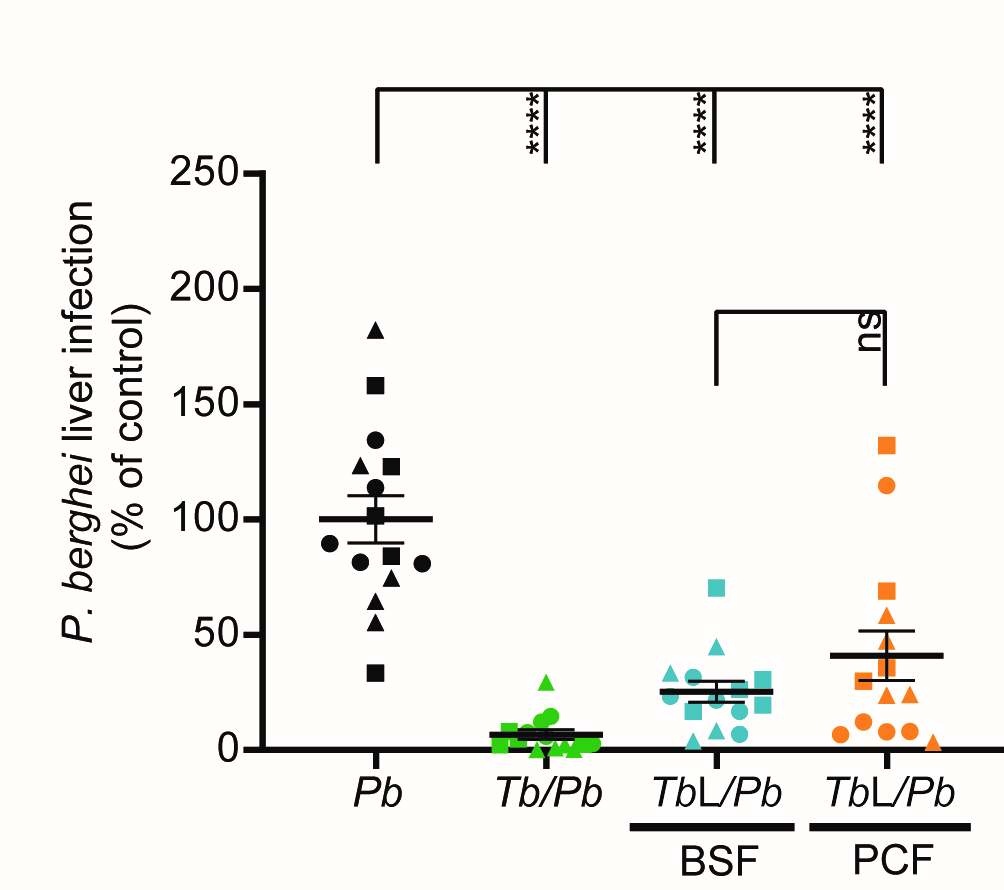

Supplement: S4 Fig — P. berghei liver infection load quantified by qRT-PCR 6h and 46 h after injection of P. berghei sporozoites into naïve mice (Pb–black symbols), mice infected 5 days earlier with T. brucei (Tb/Pb–green symbols), or mice that received lysates of either bloodstream form trypanosomes (BSF TbL/Pb—blue bars) or procyclic form trypanosomes (PCF TbL/Pb—yellow symbols) 30 min prior to sporozoite inoculation. Symbols represent the individual values of each mouse in three independent experiments, in which each symbol format corresponds to an individual experiment, and error bars indicate the SEM. The one-way ANOVA with post-test Dunnett was employed to assess the statistical significance of differences between groups. ns, not significant and ****p<0.0001. (TIF) [file pntd.0009912.s004.tif]

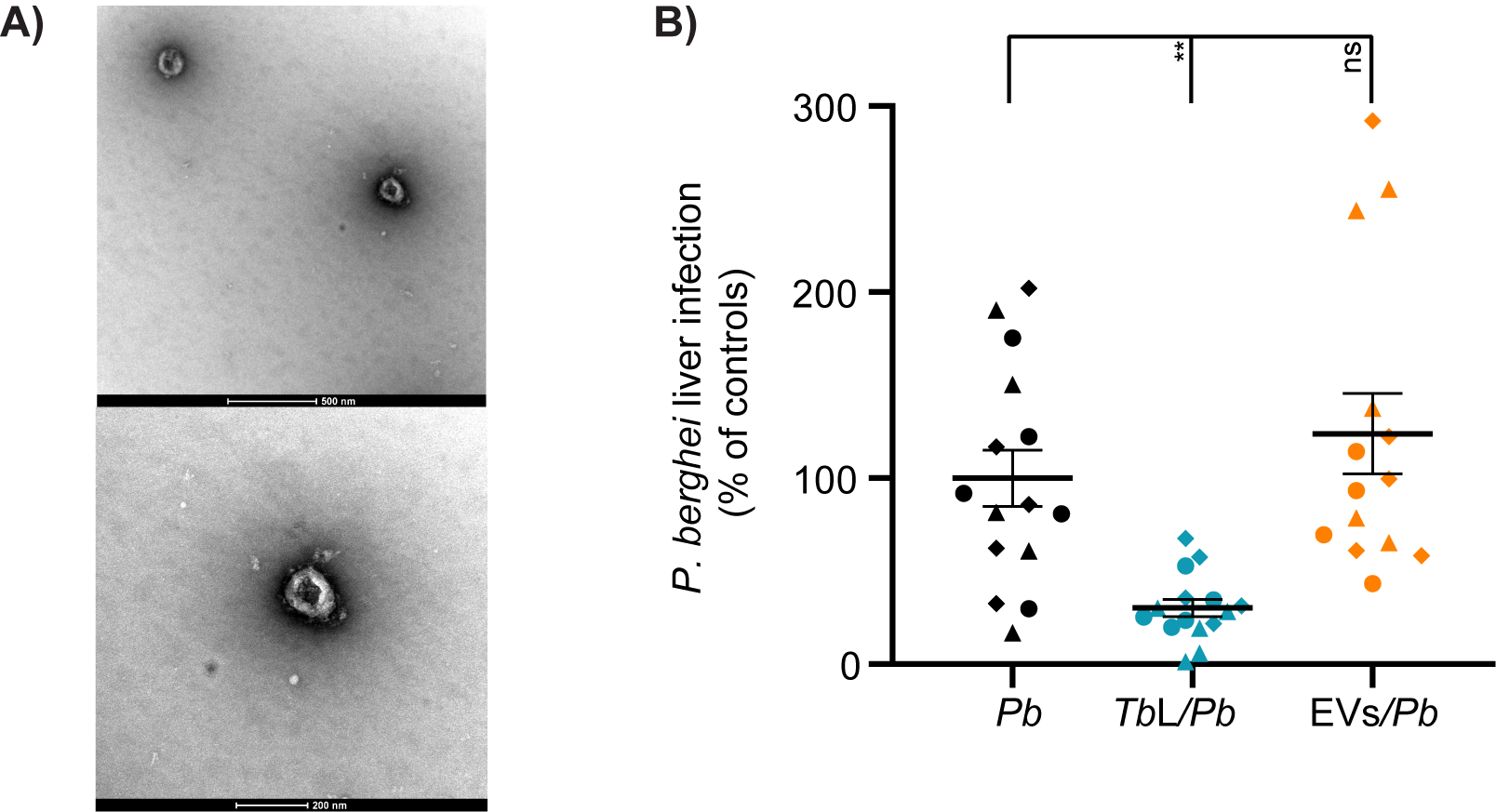

Supplement: S5 Fig — (A) TEM of purified EVs, observed by negative staining. (B) P. berghei liver infection load quantified by qRT-PCR 46 h after injection of sporozoites into naïve mice (Pb—black symbols), mice that received T. brucei total lysates (TbL/Pb—blue symbols), trypanosome EVs (EVs/Pb—orange symbols) 30 min prior to sporozoite inoculation. Symbols represent the individual values of each mouse of three independent experiments and error bars indicate the SEM. The one-way ANOVA with post-test Dunnett was employed to assess the statistical significance of differences between groups. ns, not significant and **p<0.01. (TIF) [file pntd.0009912.s005.tif]

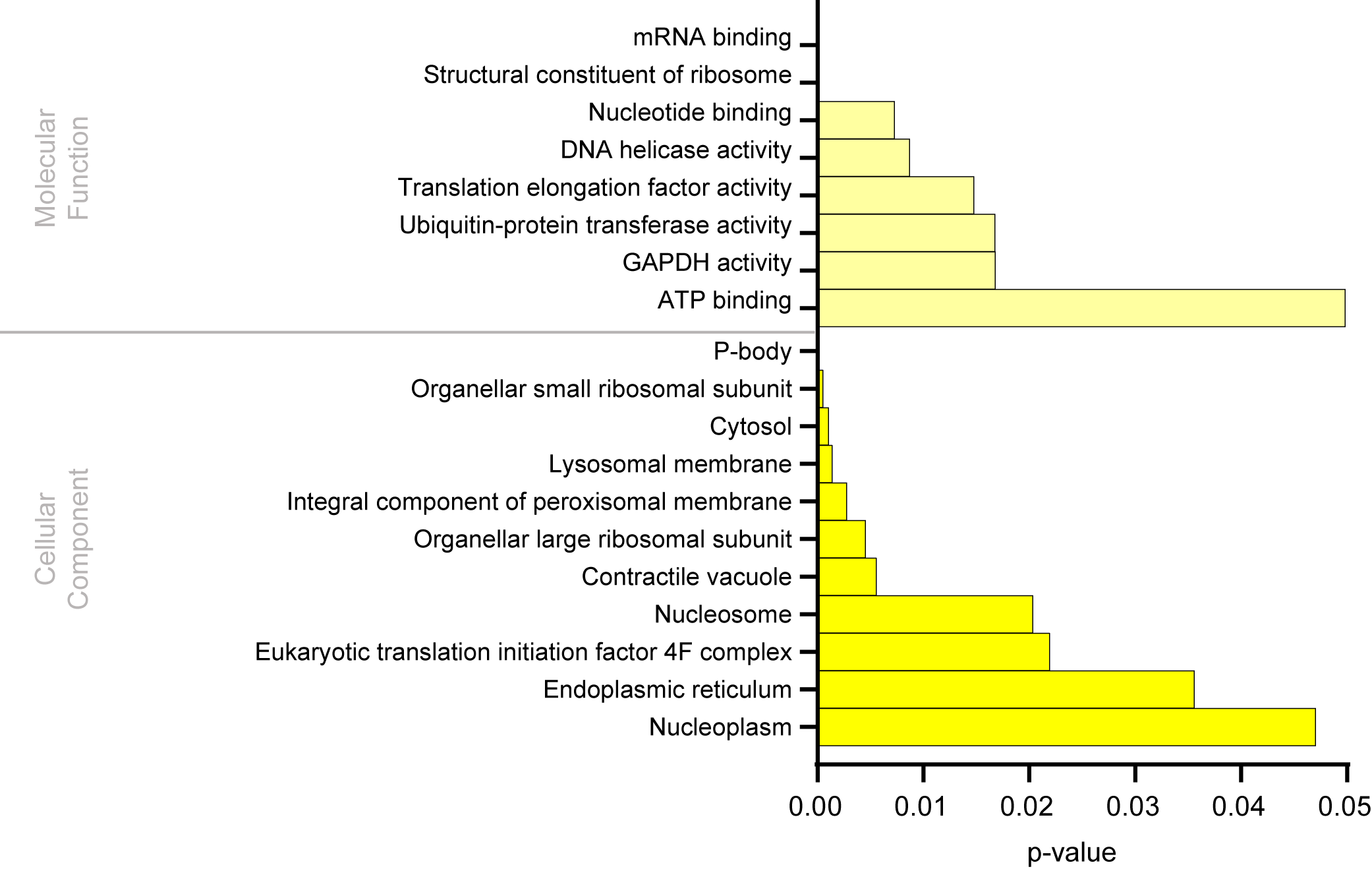

Supplement: S6 Fig — GO categories included: molecular function and cellular component. (TIF) [file pntd.0009912.s006.tif]
