# Supplementary material for: Excreted Trypanosoma brucei proteins inhibit Plasmodium hepatic infection
Source: PLoS Negl Trop Dis. 2021 Oct 29;15(10):e0009912. doi: 10.1371/journal.pntd.0009912 (PMC8580256; doi:10.1371/journal.pntd.0009912)
Supplement: Attachment — Submitted filename: renamed_ae27e.docx [file pntd.0009912.s009.docx]

**Reviewer #1: This clearly defined and well written study describes the inhibition of Plasmodium berghei liver infection following passive transfer from mice previously infected by T. brucei, and with total protein lysates from T. brucei. In depth and logical biochemical analysis demonstrates that protective efficacy in independent of VSG levels, and that GPI aochored proteins are not responsible for mediating protection. Initial proteomic analysis was then performed to identify the inhibitory trypanosomal components, with mass spec identifying 376 proteins present to potentially contribute to protection, 127 which have been previously defined in the T. brucei secretome.**

**This work builds largely on previously work described in Sanches-Vas et al., 2019, and Eyford et al., 2013 – and is a logical and meaningful extension to these findings. Experiments are well designed and described, with appropriate controls. Conclusions are supported by the results, and referencing is correct throughout. Samples sizes, ethics and stats are all correct.**

We thank the reviewer for her/his summary of our results and for the positive remarks about our work.

**Reviewer #2: Methods are fine - if not exhaustive.**

**Results
Reviewer #1: Analysis is correct, and results are clearly and completely presented. All figures are appropriate, with one suggested edit (below).**

**Reviewer #2: Also okay (see general comments below)**

**Conclusions

Reviewer #1: Conclusions are  supported by data - I would like a brief expansion on public health relevance, but this is a minor point.**

The present study reinforces the importance of understanding the impact of co-infections, since it may have important repercussions on human and animal health. Overall, our results unveiled that during a *T. brucei* infection parasitic excreted proteins are likely released into and circulate in the bloodstream of the infected host, protecting it against a subsequent hepatic infection by *Plasmodium* parasites. Future unravelling of the mechanism behind this phenotype may contribute to a deeper understanding of *Plasmodium* biology, and possibly to the development of novel, urgently needed, antiplasmodial intervention strategies. This point has now been added to the Discussion (line 826).

**Reviewer #2:** Not sure these are justified. See comments below.

**Editorial and Data Presentation Modifications?**

**Reviewer #1: Figure 1G – would it be possible to make the Tb/Pb line a different colour? Very easy to miss at the moment.**

We have changed the colour of the Tb/Pb line in Figure 1G, as well in all the other figures, as requested by the reviewer.

**Reviewer #2:** (No Response)

**Summary and General Comments**

**Reviewer #1: This clearly defined and well written study describes the inhibition of Plasmodium berghei liver infection following passive transfer from mice previously infected by T. brucei, and with total protein lysates from T. brucei. In depth and logical biochemical analysis demonstrates that protective efficacy in independent of VSG levels, and that GPI anchored proteins are not responsible for mediating protection. Initial proteomic analysis was then performed to identify the inhibitory trypanosomal components, with mass spec identifying 376 proteins present to potentially contribute to protection, 127 which have been previously defined in the T. brucei secretome.**

**This work builds largely on previously work described in Sanches-Vas et al., 2019, and Eyford et al., 2013 – and is a logical and meaningful extension to these findings. Experiments are well designed and described, with appropriate controls. Conclusions are supported by the results, and referencing is correct throughout. Samples sizes, ethics and stats are all correct.**

**I have a few minor edits to suggest:**

**1). Line 133 refers to infection by retro-orbital route – why is this chosen as an infection route? i.v is not entirely representative of natural infection (as the authors state here – 5 infectious bites is standard and easily performed)?**

Sanches-Vas *et al.*, 2019 described that the *Anopheles* mosquitoes showed a feeding preference for *T. brucei*-infected animals relative to uninfected mice, leading to high experimental variability between different groups of animals. Also, since the number of sporozoites injected is likely to vary between mosquitoes, introducing additional variability in the inoculum of different animals. Thus, the i.v. administration of defined numbers of sporozoites enables the normalization the number of parasites inoculated, while mosquito bites can lead to a much higher variability between animals. Additionally, the inoculation of *P. berghei* sporozoites by mosquito bite was shown to be less efficient than i.v. inoculation (S. Conteh *et al.* 2010). Finally, the sporozoite dose employed in our study was selected on the basis of previous work indicating that it constitutes an adequate surrogate of infection by 5 mosquito bites (Prudêncio M. *et al.*, 2006).

**2). Lines 375-387. A very nice set of experiments to demonstrate that impairment of infection is due to a decrease in the number of infected hepatocytes rather than impairment of intrahepatic replication. This leads to the obvious follow on question – is impairment due to effects on the sporozoite (prior to invasion), or the hepatocyte (post-invasion)? Clarity on this would be welcome – e.g. a IFA against sporozoites would at least demonstrate if serum recognises the surface of the sporozoite pre-infection.**

We thank the reviewer for this interesting and pertinent remark. We would like to point out that the timeline of our experiment is too short for animals to develop an adaptive immune response (2 days between sporozoite inoculation and the end of invasion). It is also extremely unlikely that the injection of *T. brucei* lysates would increase the levels of polyreactive antibodies in such a short period of time. Thus, it is extremely improbable that the serum of an animal injected with lysates may contain antibodies that recognize the surface of the sporozoite.

However, your point on whether the effect is prior or post-invasion is certainly a valid one. Our data is compatible with an effect of lysates both prior and post-invasion by the sporozoites. The data shown in figure 2C demonstrates that injection of the *T. brucei* lysates after the infection is established (6h post-infection) induces a marked reduction in *P. berghei* liver load, suggesting a strong effect on the infected hepatocyte (post-invasion). Nonetheless, this inhibitory effect is somewhat less pronounced than that observed for *T. brucei* lysates injected 30 min prior to *P. berghei* inoculation, suggesting that the protection conferred by the *T. brucei* lysates may also act on the sporozoite (prior to invasion). This point has now been added to the Discussion (line 774).

**3). Figure 1G – would it be possible to make the Tb/Pb line a different colour? Very easy to miss at the moment.**

We have changed the colour of the Tb/Pb line in Figure 1G, as well in all the other figures, as requested by the reviewer.

**4). Lines 454-456 – you conclusively show that protection is time dependent – how does the data you see compare to the natural clearance rate of other proteins in mice after passive transfer (e.g. – of transfused IgG)? Does the protective effect decrease due to the standard kinetics of protein clearance in mice?**

The reviewer raises an interesting point. The protective effect of *T. brucei* lysates decreases not only due the kinetics of protein clearance in mice, but also in accordance to the half-life of the protein(s) responsible for the observed phenotype. The half-life of each specific protein is different, for example, in mouse serum, the half-life of IgG is of 95 h, while albumin has a half-life of 35 h (Chaudhury C. *et al.*, 2003), in tissues, the half-life of the enzyme ornithine decarboxylase can range from 5 to 30 minutes (Iwami K., et al. 1990). However, it has also been shown that the lifespan of a protein may vary between tissues, as protein turnover in the brain is slower (average half-life of 9 days) when compared to the blood (3.5 days) or the liver (3 days) (Price J., *et al.* 2010). This point has now been added to the Discussion (line 815).

**Reviewer #2: This paper is a followup to the work of M. Vas, M. Prudencio and L. Figueiredo ("Trypanosoma brucei infection protects mice against malaria", PNAS 2019). Here, the authors drill down to try and identify the substance, released by T.brucei, that blocks infection by plasmodiumm. Toward this, they show that protection from hepatocyte infection can be conferred by:
- passive transfer of serum from T.brucei infected animals
- injection with T.brucei lysates (in a dose dependent manner and without overt toxicity) and only if administered closely enough to inoculation with plasmodium. This very short time window already suggests that protection is not dependent on the development of an immune response (which they also then formally demonstrate in Fig4)but rather dependent on a substance secreted or excreted by the parasite.**

We thank the reviewer for this summary of our study.

**The mechanism by which this suppression of infectivity takes place is entirely unclear. I strongly suspect that one of the alternative possibilities offered in the discussion (that trypanosome fragments enter and decorate the surface of the hepatocyte thus somehow altering its properties and rendering it more resistant to plasmodium infection) is in fact correct. This is the possibility that material found in serum and in lysates - but which is particulate (like EVs secreted by Tbrucei - decorated with VSG but wholly different than soluble VSG which the authors also test) could in fact incorporate into hepatocyte membranes when injected (as was already shown for RBC membranes by Szempruch et al (PMID: 26771494). This is an experiment that's straightforward to do and could solve the mystery (the experiment would be: generate EVs,e.g. from VSG2 expressing Tbrucei, ideally in PLC-/- background;  see if they incorporate using anti-VSG2 antibody to chase the signal; then go
backward and search for EVs in lysates and serum of infected animals).**

We thank the reviewer for this interesting suggestion. In accordance with this, we purified extracellular vesicles (EVs) from *T. brucei* cultures, by ultracentrifugation. We then used transmission electron microscopy images to validate the purification of the EVs. Each mouse was injected with EVs excreted by 1x10^8^ trypanosomes, 30 min prior to *P. berghei* sporozoite inoculation. Quantification of *Plasmodium* liver load showed that administration of *T. brucei* EVs did not reduce *P. berghei* liver infection relative to that of control mice, which indicates that *T. brucei* EVs are not involved in the phenotype under study. This data is now included in Fig. S5 of the manuscript. Nevertheless, the hypothesis that proteins excreted by trypanosome, but not present in EVs can enter or/and decorate the surface of the hepatocyte, rendering it more resistant to *Plasmodium* infection, remains a possibility that cannot be excluded.
